# Small RNA Profiles of the Rice PTGMS Line Wuxiang S Reveal miRNAs Involved in Fertility Transition

**DOI:** 10.3389/fpls.2016.00514

**Published:** 2016-04-20

**Authors:** Hongyuan Zhang, Jihong Hu, Qian Qian, Hao Chen, Jing Jin, Yi Ding

**Affiliations:** State Key Laboratory of Hybrid Rice, Department of Genetics, College of Life Sciences, Wuhan UniversityWuhan, China

**Keywords:** rice PTGMS, male sterile, miRNA, fertility transition, RNA-seq

## Abstract

MicroRNAs (miRNAs) play key roles in the regulation of plant growth and developmental processes. In this study, RNA-seq was used to examine the expression profiles of miRNAs in a novel, photo-thermosensitive genic male sterile (PTGMS) rice line, Wuxiang S (WXS), during fertility transition. A total of 497 known miRNAs and 273 novel miRNAs were identified. In a differential expression analysis, 26 miRNAs exhibited significant differential expression between WXS (Sterile, S) and WXS (Fertile, F). Some of these miRNAs were validated by quantitative real-time PCR. Among these miRNAs, 11 showed decreased expression levels, and 15 showed increased expression levels in WXS (S) compared to WXS (F). Some of these miRNAs, such as osa-miR156a-j, osa-miR164d, and osa-miR528, were shown to be negatively correlated with their targets. These targets have previously been reported to be related to pollen development and male sterility, suggesting that these miRNAs may be involved in the regulation of pollen development in the rice PTGMS line WXS. Furthermore, miRNA-mediated editing events were also observed. In this study, a possible model for the control of signaling pathways during the process of fertility transition in the rice PTGMS line WXS by miRNAs was developed. These findings contribute to our understanding of the roles of miRNAs during anther development in PTGMS lines in rice.

## Introduction

MicroRNAs (miRNAs) are endogenous, small (20–24 nt), non-coding RNAs that play important roles in various biological and metabolic processes, including development, signal transduction, and biotic and abiotic stress responses (Bartel, 2004, 2009). In plants, primary miRNA transcripts (pri-miRNA) are mainly transcribed by RNA polymerase II and subsequently cleaved by Dicer-like1 (DCL1), resulting in mature miRNA sequences (Voinnet, 2009). Mature miRNAs then enter into the RNA-induced silencing complex (RISC) and negatively regulate gene expression at the post-transcription or translation level by degrading target mRNAs or by translational repression (Bartel, 2004).

A deep-sequencing study of the transcriptome detected more than 100 miRNAs in plant male gametophytes during development (Le et al., 2011). Recently, differential expression patterns of miRNAs between the cytoplasmic male sterility (CMS) line and its maintainer line have been reported in maize (Shen et al., 2011), cotton (Wei et al., 2013), *Brassica juncea* (Yang et al., 2013), cabbage (Wei et al., 2015), and rice (Yan et al., 2015). Additionally, it has been found that miRNA172 can control flowering time by down-regulating APETALA2-like target genes in Arabidopsis (Chen, 2004). Moreover, in Arabidopsis, miR167 overexpression has been reported to lead to male fertility defects (Sire et al., 2009), whereas miR159a overexpression results in decreased expression of MYB33, leading to male sterility and flowering time delays in Arabidopsis (Achard et al., 2004). Other studies have also reported that MYB33/MYB65 facilitates Arabidopsis anther development. These mutants were conditionally sterile but exhibited increased fertility under greater light levels or lower temperatures (Millar and Gubler, 2005). In photoperiod- and thermo-sensitive genic male sterile (PTGMS) rice lines such as PA64S and NK58S, a 21-nt small RNA (sRNA, osa-smR5864w) is produced by a non-coding RNA precursor *p/tms12-1*. A C-to-G substitution in the small RNA leads to loss-of-function, which gives rise to the male sterility found in PA64S and NK58S (Ding et al., 2012; Zhou et al., 2012, 2014). However, our knowledge of the involvement of miRNAs in rice PTGMS lines is still incomplete.

Hybrid rice breeding has made a tremendous contribution to food security in China (Cheng et al., 2007; Peng et al., 2008), and the utilization of male sterility in hybrid rice technologies is mainly based on three-line and two-line systems. The three-line system includes the CMS line, a maintainer line that maintains the sterility of the CMS line, and a restorer line that restores the fertility of the CMS line in hybrid rice. However, in the two-line system, the fertility of the male sterile line is influenced by the alteration of environmental conditions (day-length and temperature); thus, the male sterile line is called an environmentally sensitive genic male sterile (EGMS) or a PTGMS line. These male sterile lines can be used not only as male-sterile lines but also as maintainer lines depending on environmental factors. Therefore, the application of a two-line breeding system is simple, inexpensive, labor-saving, and effective and overcomes the limitations of the three-line system in hybrid rice (Liu et al., 2001; Yang et al., 2007; Xu et al., 2011; Zhou et al., 2012). Recently, we developed a novel male sterile line Wuxiang S (WXS) for use in two-line systems. This line was derived from a mutant tms5 locus in *indica* rice. In this study, we used RNA-seq to identify conserved and novel mircoRNAs that may be involved in fertility transition during pollen development in WXS. The interaction network between these miRNAs and their targets was also investigated using GO and KEGG analysis, and we attempted to elucidate the potential regulatory mechanism of pollen development during fertility transition in the rice PTGMS line WXS.

## Materials and methods

### Rice materials

The rice PTGMS line WXS was generated by our laboratory. Between May and August 2014, WXS plants were grown in the natural ecological paddy field of Huazhong Agricultural University (30°28′ N, 114°21′ E), Wuhan, Hubei province of China. From 20 July to 3 August 2014, natural ecological conditions were daily average temperatures between 25.5°C and 34.6°C, with approximate day lengths of 14 h light and 10 h dark in Wuhan (Wuhan Weather Bureau), which could induce WXS sterility, and no pollen was produced in male sterile rice, a condition designated as WXS (S). When the young panicle length was approximately 1 cm, we transferred 20 of these WXS plants from the paddy field into a cooling pond to treat them with lower temperatures (approximately 21°C) and shorter day lengths (approximately 12 h light/12 h dark) for 2 weeks. These plants were converted into male-fertile plants, designated WXS (F). Young panicles were separately collected from the natural ecological paddy field and from the cooling pond at the pollen mother cell (PMC) formation stage (P2) and the meiosis stage of PMC (P3) and were correspondingly named SP2, SP3, FP2, and FP3. Collected samples were frozen in liquid nitrogen and then stored at −80°C for future use. To analyze pollen fertility, mature anthers and pollen grains were also collected and stained with 1% potassium iodide solution (I_2_-KI). PMCs were stained using carbol fuchsin dye for cytological observations. Rice anther staging was performed in this study according to the method of Itoh et al. (2005).

### RNA isolation, small RNA library construction, sequencing, and data analysis

Total RNA was extracted from the young rice panicles at the PMC and meiosis stages using Trizol reagent (TaKaRa, Dalian, China) according to the manufacturer's instructions. RNA quality and quantity were measured using a NanoDrop2000 and Agilent 2100 bioanalyzer. Then, small RNA libraries were constructed and analyzed as described by Molly and Thomas (2010). Briefly, small RNAs ranging from 18 to 30 nt were size fractionated using 15% polyacrylamide gel electrophoresis (PAGE), and suitable isolated fragments were extracted from the gel and ligated with 5′ and 3′ RNA adaptors using T_4_ ligase. Subsequently, the resulting products were reverse transcribed and amplified using 15 cycles of PCR to produce sequencing libraries. Finally, flow cell sequencing was performed on the HiSeq 2500 platform. Automated base calling of the raw sequences and vector removal were performed with the PHRED and CROSS MATCH programs.

After Illumina sequencing, any low quality reads, adaptors, contaminating sequences and sequences shorter than 18 nt were discarded. Only the remaining high-quality sequences between 18 and 30 nt were further analyzed. All unique sequences were aligned to the rice genome (Nipponbare-Reference- IRGSP-1.0) and annotated based on MSU-v7.0 (ftp://ftp.plant biology.msu.edu/pub/data/Eukaryotic_Projects/o_sativa/annotation_dbs/) using SOAP for mapping (Li et al., 2008). Reads that mapped to rice rRNA, tRNA, scRNA, snRNA, or snoRNA were removed based on the National Center for Biotechnology Information (NCBI) (http://www.ncbi.nlm.nih.gov/) and Rfam RNA family databases (Gardner et al., 2009). Known miRNAs were identified using a BLAST search against the miRNA database miRBase release 20 (http://mirbase.org/) (Kozomara and Griffiths, 2011). Reads that did not annotate to any category were used to predict novel miRNAs using the miRNA prediction program MIREAP (http://sourceforge.net/projects/mireap/). The method for selecting potential miRNAs or pri-miRNAs was as described by Yan et al. (2015). Secondary structures of potential miRNA precursors were constructed using the MFOLD3.2 web server (http://mfold.rna.albany.edu/; Zuker, 2003).

### Differential expression analysis of miRNAs

To identify miRNAs with differential expression in the four samples (SP2 and FP2, SP3 and FP3), miRNA read counts were normalized to transcripts per million (TPM) using the following formula: normalized expression = (miRNA count/total count of clean reads) × 10^6^. If one miRNA was not expressed in one of two samples, the normalized read count of this miRNA was arbitrarily set as 0.01 for further calculation (Chen et al., 2012). When miRNA expression levels were less than 1 TPM in both samples, differential expression analysis was not performed. Fold changes and *P* values were calculated to determine the significance of expression differences between SP2 and FP2 and between SP3 and FP3. Hierarchical clustering and k-means clustering of the expression patterns were performed in Mutiexperimental Viewer v4.7 (Saeed et al., 2006).

### Prediction of the miRNA targets and functional analysis

The sequences of all known and novel miRNAs were aligned to the annotated rice genome (MSU-v7.0) to predict potential target genes. The criteria used for target prediction in this study were as described by Hu et al. (2014). The potential targets of known and novel miRNAs that were significantly differentially expressed between two samples (SP2-FP2 or SP3-FP3) were searched against the Gene Ontology database and KEGG databases (Kanehisa and Goto, 2000). The biological process, molecular function and cellular component of the targets were obtained, and then GO functional enrichment analysis was performed using AgriGO with *Oryza sativa* MSU-v7.0, and graphical results depicting overrepresented GO terms were generated via singular enrichment analysis to fully understand the function of these targets.

### miRNA detection by stem-loop RT-PCR and validation by qPCR

After total RNA was extracted from the young panicles at the PMC and meiosis stages, RNA-free DNaseI (Fermentas, USA) was used to remove DNA contamination for 30 min at 37°C. Approximately 2 μg of total RNA was reverse transcribed using miRNA-specific stem-loop primers and a Fermentas RevertAid First Strand cDNA Synthesis Kit (Fermentas, USA), as described previously (Varkonyi et al., 2007). The reactions were incubated for 30 min at 16°C; followed by 60 cycles of pulsed reverse transcription at 30°C for 30 s, 42°C for 30 s and 50°C for 1 s; and finally terminated by incubating at 70°C for 5 min. For miRNA targets, cDNA templates were reverse transcribed using the Oligo dT_18_ primer with a Fermentas RevertAid First Strand cDNA Synthesis Kit (Fermentas, USA).

Expression analysis of the known miRNAs and their potential targets was performed using the ABI Step One Plus™ Real Time PCR System (Applied Biosystems, USA) and a SYBR Green Master Mix (Roche, Germany). Quantitative real-time PCR (qPCR) was performed using the following parameters: 10 min at 95°C, followed by 40 cycles of 15 s at 95°C and 60 s at 60°C. U6 snRNA and *OsActin* were chosen as endogenous controls for miRNAs and targets, respectively. The reactions were performed with three biological replicates, and a melting curve analysis was carried out to verify that only one specific amplification occurred. Comparative expression levels were calculated in the four different samples using the 2^−▵▵*CT*^ method (Livak and Schmittgen, 2001). Novel miRNAs were detected by electrophoresis analysis after stem-loop RT-PCR. All of the primers used in this study are listed in Table S1.

### Identification of potential miRNA editing sites and validation of these sites

The RNA editing level was calculated by the ratio of reads supporting the mismatch in a site to the total reads detected at that site. MiRNA editing sites located on known miRNA sequences were identified according to the methods described by Luciano et al. (2004) and Yang et al. (2013). Genomic DNA and total RNA were isolated from young panicles to validate miRNA editing. Precursor miRNA sequences were amplified from rice DNA, and the corresponding mature miRNA sequences were also amplified from cDNA, which was reverse-transcribed using stem-loop RT-PCR. All primers used in this study are listed in Table S1.

## Results

### Cytological observation of WXS under fertility transition

**Figure 1:**
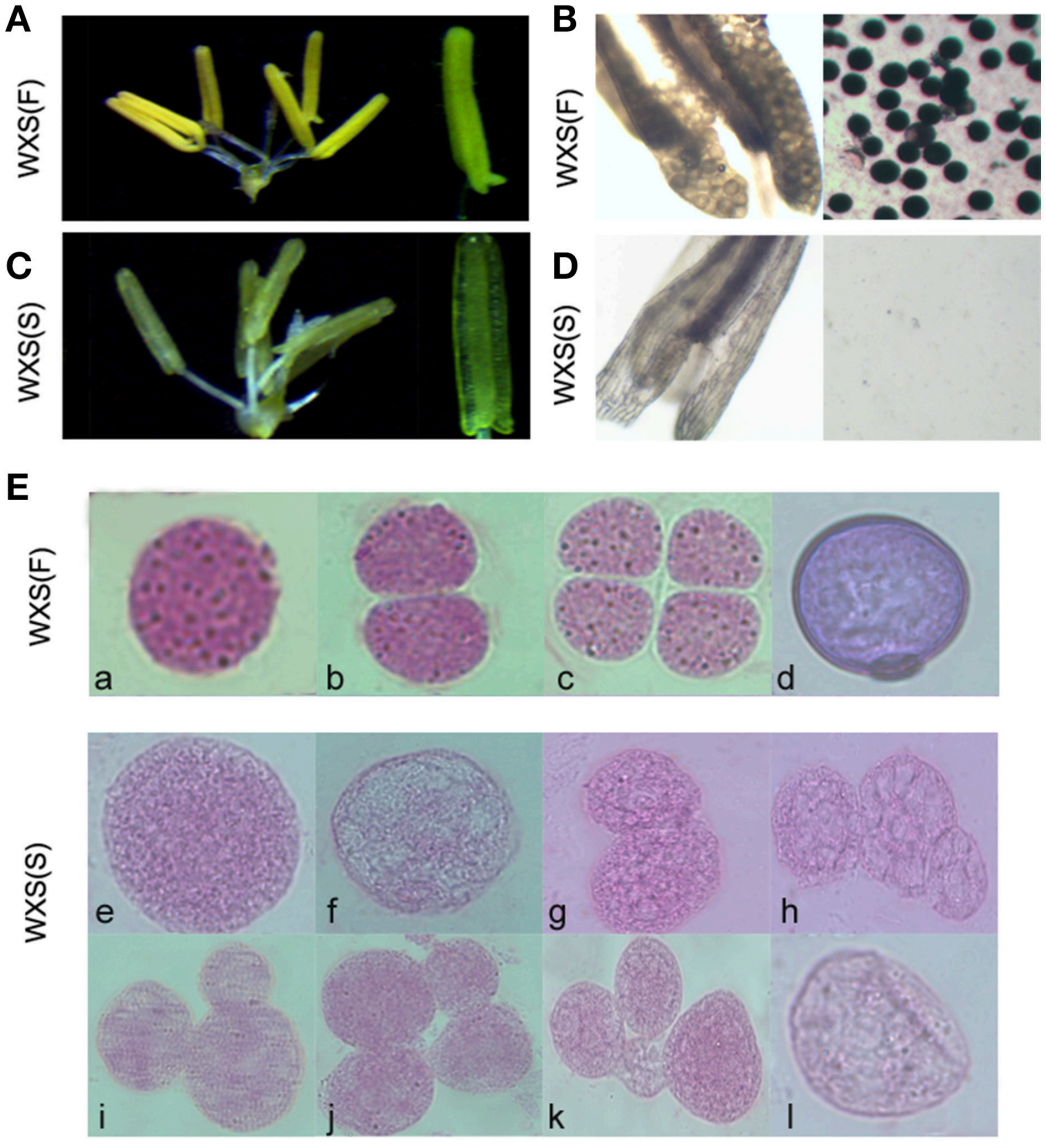
**Fertility observation in the process of fertility transition for WXA. (A)** Mature, normal anthers of WXS (F) observed by stereo microscope. **(B)** Mature anthers of WXS (F) stained darkly with 1% potassium iodide solution (I_2_-KI). **(C)** Mature anthers of WXS (S) observed by stereo microscope. **(D)** Mature anthers of WXS (S) stained with 1% potassium iodide solution (I_2_-KI). Carbol fuchsin dyeing of the WXS (S) **(E,a–d)** and WXS (F) **(E,e–l)** microsporocytes from the pollen mother cell formation stage to the pollen meiosis stage. **a, e, f** were considered the pollen mother cell formation stage; **b, c, g, h, i, j, k** were considered the meiosis stage; **d, l** were considered the mononuclear stage.

WXS was grown in the paddy field until the panicle length was approximately 1 cm, and then 20 plants were selected and transferred to a cooling pond to treat them with low temperatures for 2 weeks. Under the low temperature treatment (~ 21°C), WXS was male-fertile with normal anthers (Figure 1A). Pollen grains from these samples were stained using a 1% I_2_-KI solution (Figure 1B). Fertile pollen grains maintained the normal morphology from pollen mother cell formation (Figures 1E,a) to mature pollen grains (Figures 1E,b–d). In contrast, under natural conditions, the WXS plants had thin anthers (Figure 1C) and a complete absence of I_2_-KI-stained pollen (Figure 1D), exhibited sterility with abnormal pollen mother cells (Figures 1E,e,f) and aberrant dyads and tetrads during meiosis (Figures 1E,g–k), and eventually produced cracked pollens (Figures 1E,l). These results suggested that the male sterile line WXS can display two different phenotypes under two different environmental conditions; these were named WXS (S, Sterile) and WXS (F, Fertile).

### Overview of small RNA library sequencing data

To identify the roles of miRNAs in the process of fertility transition during anther development in WXS, four small RNA libraries (SP2, SP3, FP2, and FP3) were constructed for deep sequencing. A total of 9,114,807, 6,775,838, 22,076,091, and 9,560,446 raw reads were generated via Illumina sequencing from the SP2, SP3, FP2, and FP3 libraries, respectively (Table S2). After removing the low quality reads, the 5′ and 3′ adapter nulls, the insert nulls, 5′ adapter contaminates, reads smaller than 18 nt, and polyA reads, a total of 8,252,111 (SP2), 5,851,620 (SP3), 17,869,357 (FP2), and 7,798,691 (FP3) clean reads were obtained (Table S2). Approximately 65.06, 65.25, 69.46, and 69.86% of the total small RNA sequences corresponding to SP2, SP3, FP2, and FP3, respectively, were mapped to the rice genome using SOAP according to the method of Li et al. (2008) (Table 1). Almost every RNA category, including miRNA, siRNA, rRNA, snoRNA, snRNA, and tRNA, was detected in the four libraries (Table 1). The results showed that known miRNAs accounted for approximately 5.94, 5.10, 6.56, and 4.80% of the sequence reads in the SP2, SP3, FP2, and FP3 libraries, respectively (Table 1).

**Table 1:** **Distribution of the small RNAs among different categories in this study**.

**Category**	**SP2**		**SP3**		**FP2**		**FP3**	
	**Unique sRNAs (%)**	**Total sRNAs (%)**	**Unique sRNAs (%)**	**Total sRNAs (%)**	**Unique sRNAs (%)**	**Total sRNAs (%)**	**Unique sRNAs (%)**	**Total sRNAs (%)**
Total clean	3350977(100)	8252111(100)	2350672(100)	5851620(100)	5826790(100)	17869357(100)	2778339(100)	7798691(100)
match_genome	1777926(53.06)	5369008(65.06)	1271845(54.11)	3818442(65.25)	3030233(52.10)	12412727(69.46)	1496007(53.85)	5447919(69.86)
rRNA	35954(1.07)	291320(3.53)	36865(1.57)	348681(5.96)	92519(1.59)	1467168(8.21)	57552(2.07)	705550(9.05)
snRNA	2869(0.09)	7661(0.09)	3198(0.14)	9753(0.17)	7951(0.15)	41285(0.23)	4523(0.16)	16487(0.21)
snoRNA	10534(0.31)	61641(0.75)	11172(0.48)	68636(1.17)	29936(0.51)	413198(2.31)	13070(0.47)	85048(1.09)
tRNA	10270(0.11)	443622(5.38)	11353(0.48)	486892(8.32)	22705(0.39)	962104(5.38)	17140(0.62)	830936(10.65)
miRNA	3704(0.11)	490177(5.94)	3039(0.13)	298197(5.10)	5278(0.09)	1172662(6.56)	3263(0.12)	374358(4.80)
No_annotation	3287646(98.11)	6957690(84.31)	2285045(97.21)	4639461(79.29)	5668401(97.28)	13812940(77.30)	2682791(96.56)	5786312(74.20)

**Figure 2:**
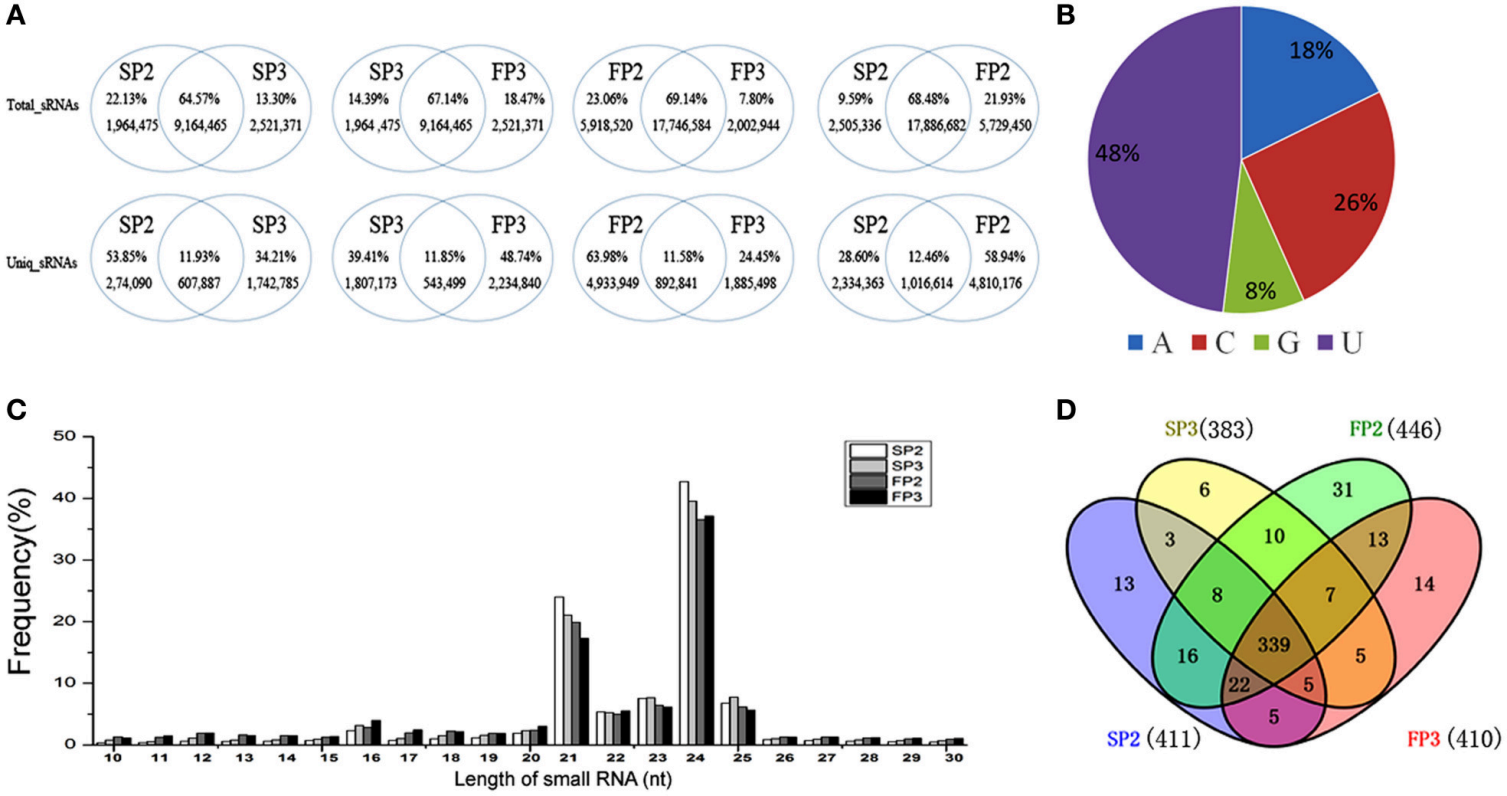
**Overview of the small RNA library sequencing. (A)** Comparisons between the common and specific reads of the small RNAs in the four libraries. (**B)** The distribution of first nucleotide of miRNAs. **(C)** The length distribution of known miRNAs. **(D)** The known miRNAs shared by all four RNA libraries.

When the common and unique reads of these small RNAs were compared between the four libraries, more than 64% of the total sRNAs were shared when any two libraries were compared: 64.58% were shared between SP2 and SP3, 67.14% were shared between SP3 and FP3, 68.48% were shared between SP2 and FP2, and 69.14% were shared between FP2 and FP3 (Figure 2A). Correspondingly, more than 11% of unique small RNAs were found when any two libraries were compared, suggesting that there was a less abundant but much more diverse pool of small RNAs that could be assumed to represent induced-specific small RNAs (Figure 2A). These data emphasize the differences and complexities in the assemblages of small RNAs between the different environments in the process of fertility transition in WXS.

### Identification of known miRNAs

In plants, the first nucleotide of a miRNA is important for the construction of the RNA induced silencing complex (RISC). In Arabidopsis, different Argonaute (AGO) proteins are preferentially enriched for the different initiating nucleotides of miRNAs; for example, AGO1 prefers miRNAs beginning with a U, whereas AGO2 and AGO4 recruit miRNAs with a 5′ terminal A (Mi et al., 2008). In our study, we found that 48% of the first nucleotides of miRNAs were U, followed by C at 26%, A at 18%, and G at 8% (Figure 2B). These results were similar to those of a previous study in which most of the miRNAs in rice began with a U (Yan et al., 2015). To identify known miRNAs, unique small RNA sequences were aligned against mature miRNAs from rice deposited in the miRBase database (Release 20) (Kozomara and Griffiths, 2011). Currently, approximately 713 mature miRNAs derived from 592 pre-miRNAs are published in the miRBase (June 2013). In our study, the length distribution of small RNAs was similar between the four libraries, with fragment sizes mainly between 21 and 24 nt in length (Figure 2C). A total of 497 known miRNAs were identified, of which 411 miRNAs were in SP2, 446 in FP2, 383 in SP3, and 410 in FP3 (Table S3). Among these miRNAs, 339 known miRNAs were shared between all four RNA libraries, accounting for 68.21% of the identified miRNAs (Figure 2D). Moreover, a total of 64 miRNAs, of which 13 were in SP2, 31 were in FP2, 6 were in SP3, and 14 were in FP3, were detected in only one of the four samples (Figure 2D). This indicates that those miRNAs are likely to be specifically expressed in the different anther development stages in WXS.

### Identification of novel miRNAs

A large number of small RNAs generated by high-throughput sequencing were used to identify novel miRNAs. In this study, a total of 373 novel miRNAs were found, many of which had low expression levels (Table S4). Of these miRNAs, 201 miRNAs were located on the 5′ arm of the miRNA precursor, and 160 miRNAs were located on the 3′ arm; 12 miRNA^*^ sequences were also obtained (Table S4). The miRNA* sequence plays an important role in miRNA prediction. However, because most miRNA^*^s are degraded soon after their separation from their corresponding miRNAs, it is thought that miRNA^*^s are usually rather low in abundance or are even undetectable (Meyers et al., 2008).

**Figure 3:**
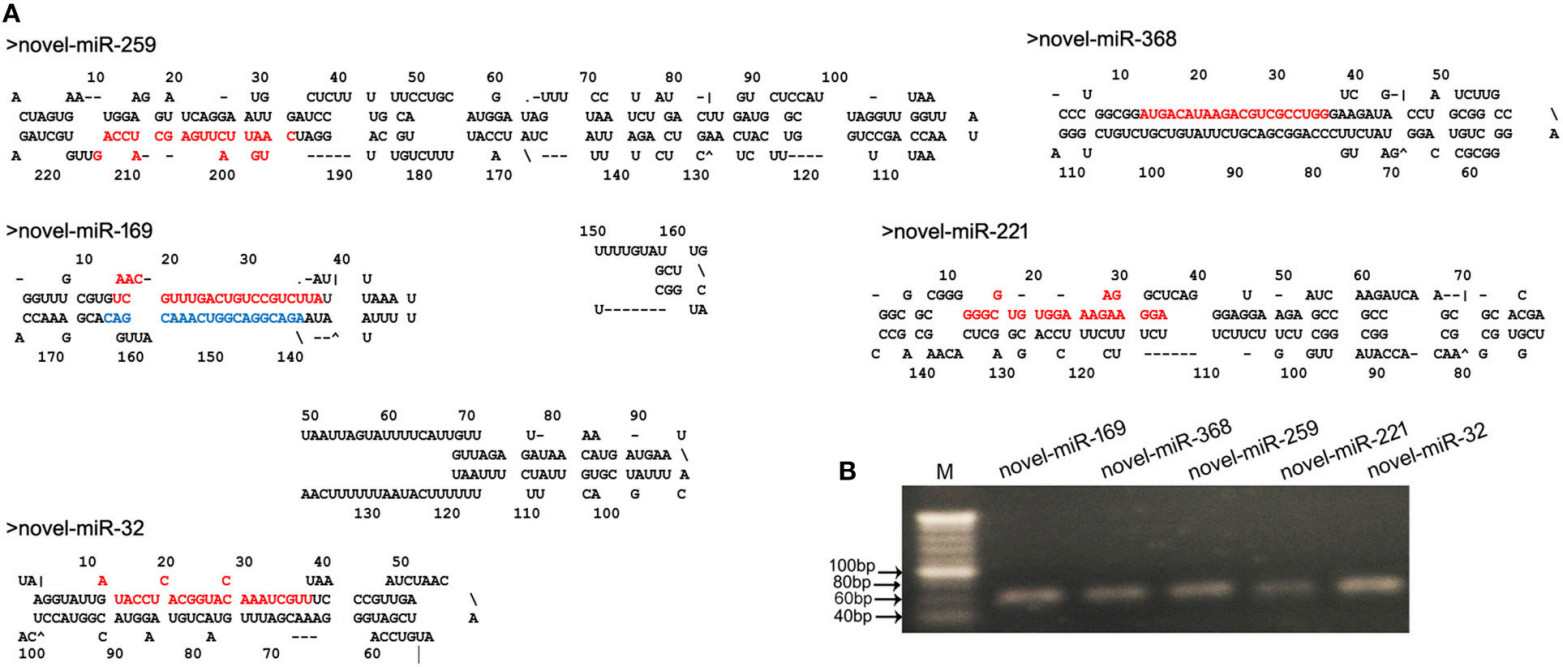
**Predicted novel miRNAs identified in this study. (A)** Predicted stem-loop structures of novel miRNA precursors. The precursor structures of five newly identified rice miRNAs (novel-miR-259, 169, 368, 221, and 32) were predicted via the MFOLD pipeline. Mature miRNA sequences are highlighted in red, and miRNA169^*^ is highlighted in blue. **(B)** Stem-loop RT-PCR analysis of the identified novel miRNAs. M indicates a 20-bp DNA Ladder Marker.

Fragment size distribution analysis indicated that most of the novel miRNAs displayed a nucleotide length of 21 nt (Figure S1). In plants, the determination of which strand of the miRNA:miRNA* duplex is incorporated into the RISC is largely based on the identity of the first nucleotide. In the present study, the first nucleotide analysis of these novel miRNAs showed that the base rates of U, C, A, and G were 49, 26, 17, and 9%, respectively (Figure S1). These novel miRNAs exhibited differential expression profiles. To validate the predicted novel miRNAs, five novel miRNAs, including novel-miR-32, novel-miR-169, novel-miR-221, novel-miR-259, and novel-miR-368 (Table S4), were selected for confirmation by stem-loop RT-PCR. PCR indicated that the DNA fragments were approximately 60-bp long. Moreover, the secondary structures of these novel miRNAs were predicted using MFOLD (http://mfold.rna.albany.edu/) and manually checked. The results showed that the five novel miRNAs had perfect secondary structures (Figure 3A). Both the PCR results and the predicted secondary structures showed that these novel miRNAs are real and reliable and revealed that these five novel miRNAs were expressed in the young panicles of WXS (Figure 3B).

### Comparative miRNA expression profiles of WXS during fertility transition

Differentially expressed miRNAs were identified in this study based on the normalization of the read counts. The relative abundances of 72 known miRNAs were found to be significantly different (^*^P < 0.05; ^**^P < 0.01) during fertility transition (SP2-FP2 (WXS (S)-WXS (F)) or SP3-FP3(WXS (S)-WXS (F)) (Table S5). Fifty-five and 51 miRNAs were significantly differently expressed in SP2-FP2 and SP3-FP3, respectively (Figure S2). Among them, expression levels of 26 miRNAs were significantly different between WXS (S) and WXS (F) (Table 2).

**Table 2:** **List of the significantly differential expressed miRNAs between the sterile line WXS(S) and fertile line WXS(F) in this study**.

**MiR-name**	**Normalized expression (TPM)**				**STD**				**Fold-change(log2)**		***P*****-value**		**Significance**	
	**FP2**	**SP2**	**FP3**	**SP3**	**FP2**	**SP2**	**FP3**	**SP3**	**SP2/FP2**	**SP3/FP3**	**SP2 vs. FP2**	**SP3 vs. FP3**	**SP2 vs. FP2**	**SP3 vs. FP3**
**DOWNREGULATED**														
osa-miR171c-5p	163	16	40	8	9.12	1.94	5.13	1.37	−2.23	−1.91	0.00	0.00	^**^	^**^
osa-miR1878	106	15	45	15	5.93	1.82	5.77	2.56	−1.71	−1.17	0.00	0.00	^**^	^**^
osa-miR156a	87	14	28	9	4.87	1.70	3.59	1.54	−1.52	−1.22	0.00	0.02	^**^	^*^
osa-miR156e	87	14	28	9	4.87	1.70	3.59	1.54	−1.52	−1.22	0.00	0.02	^**^	^*^
osa-miR156i	87	14	28	9	4.87	1.70	3.59	1.54	−1.52	−1.22	0.00	0.02	^**^	^*^
osa-miR812l	136	22	64	21	7.61	2.67	8.21	3.59	−1.51	−1.19	0.00	0.00	^**^	^**^
osa-miR156b-5p	86	14	28	9	4.81	1.70	3.59	1.54	−1.50	−1.22	0.00	0.02	^**^	^*^
osa-miR156c-5p	86	14	28	9	4.81	1.70	3.59	1.54	−1.50	−1.22	0.00	0.02	^**^	^*^
osa-miR156g-5p	86	14	28	9	4.81	1.70	3.59	1.54	−1.50	−1.22	0.00	0.02	^**^	^*^
osa-miR812m	136	24	65	21	7.61	2.91	8.33	3.59	−1.39	−1.22	0.00	0.00	^**^	^**^
osa-miR812k	153	33	77	27	8.56	4.00	9.87	4.61	−1.10	−1.10	0.00	0.00	^**^	^**^
**UPREGULATED**														
osa-miR5796	182	188	47	107	10.19	22.78	6.03	18.29	1.16	1.60	0.00	0.00	^**^	^**^
osa-miR164d	12	13	13	21	0.67	1.58	1.67	3.59	1.23	1.11	0.03	0.03	^*^	^*^
osa-miR1423-5p	2047	2425	774	1521	114.55	293.86	99.25	259.93	1.36	1.39	0.00	0.00	^**^	^**^
osa-miR444f	46	58	23	67	2.57	7.03	2.95	11.45	1.45	1.96	0.00	0.00	^**^	^**^
osa-miR159c	19	25	5	14	1.06	3.03	0.64	2.39	1.51	1.90	0.00	0.01	^**^	^**^
osa-miR159e	19	25	5	14	1.06	3.03	0.64	2.39	1.51	1.90	0.00	0.01	^**^	^**^
osa-miR159d	19	27	5	14	1.06	3.27	0.64	2.39	1.62	1.90	0.00	0.01	^**^	^**^
osa-miR530-5p	15	24	11	33	0.84	2.91	1.41	5.64	1.79	2.00	0.00	0.00	^**^	^**^
osa-miR399j	18	29	11	41	1.01	3.51	1.41	7.01	1.80	2.31	0.00	0.00	^**^	^**^
osa-miR408-3p	317	600	251	597	17.74	72.71	32.18	102.02	2.04	1.66	0.00	0.00	^**^	^**^
osa-miR528-5p	199	392	137	267	11.14	47.50	17.57	45.63	2.09	1.38	0.00	0.00	^**^	^**^
osa-miR3979-5p	36	76	13	107	2.01	9.21	1.67	18.29	2.19	3.46	0.00	0.00	^**^	^**^
osa-miR398b	166	486	105	323	9.29	58.89	13.46	55.20	2.66	2.04	0.00	0.00	^**^	^**^
osa-miR399d	17	52	10	53	0.95	6.30	1.28	9.06	2.73	2.82	0.00	0.00	^**^	^**^
osa-miR3979-3p	10	49	3	38	0.56	5.94	0.38	6.49	3.41	4.08	0.00	0.00	^**^	^**^

STD represent normalised expression level of miRNAs. Normalised expression (TPM) = count of miRNA/total count of clean sRNAs 9 × 10^6^. “_**_” and “_*_” represnent significant differential expression and significant differential expression, respectively.

**Figure 4:**
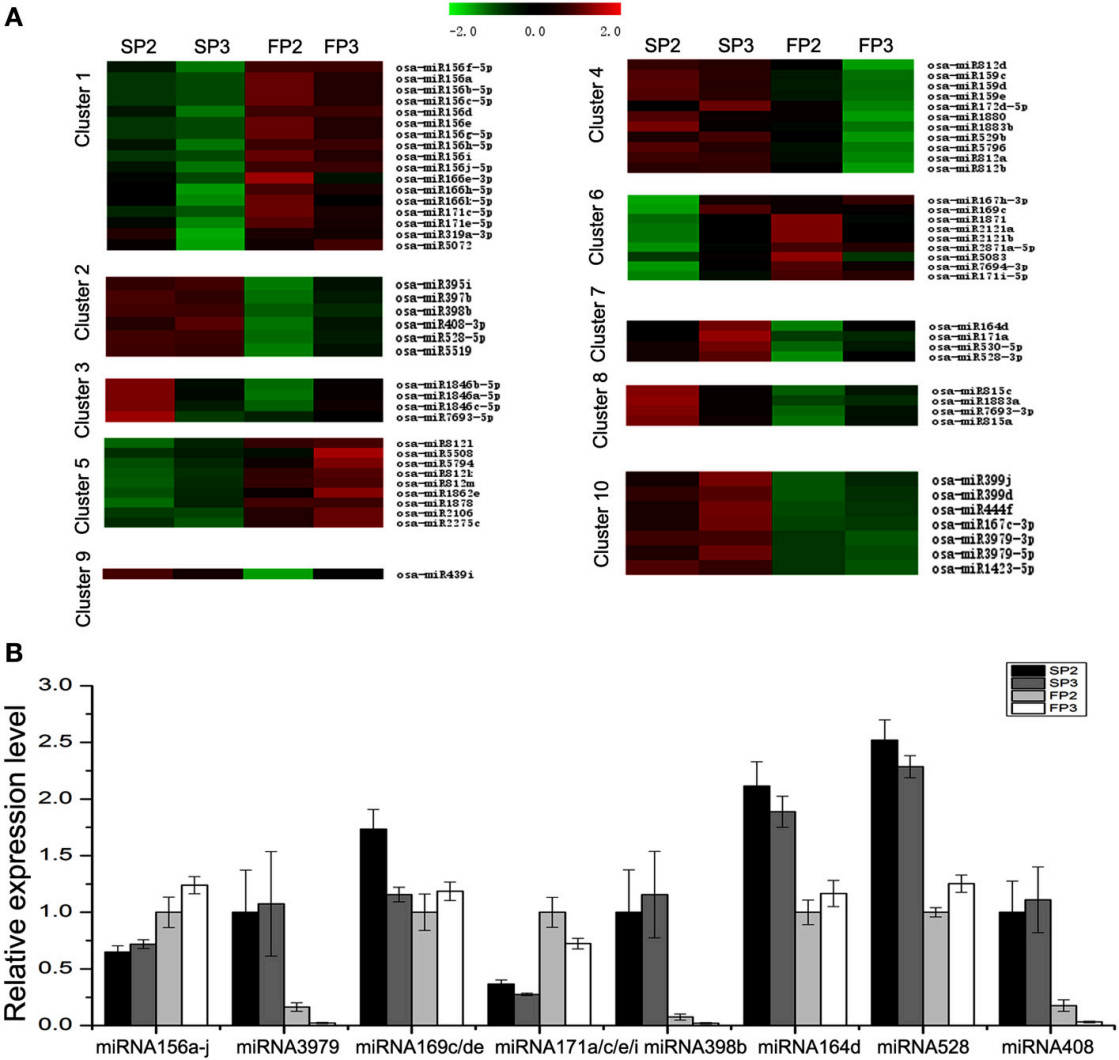
**Differential expression analyses of known miRNAs. (A)** Clustering analysis of the differentially expressed known miRNAs. **(B)** Validation via quantitative real-time RT-PCR of the differentially expressed miRNAs obtained from deep sequencing. U6 snRNA was used as a reference. The error bars indicate the standard deviations of three replicates.

In this study, 10 different clusters were obtained from Hierarchical Clustering (HCL) analysis of the expression patterns using the software MeV (Figure 4A). The results show that many miRNAs were only up-regulated in SP2 or SP3 of WXS (S) (Clusters 3, 7, 8, and 9), though some miRNAs were up-regulated in both SP2 and SP3 of WXS (S) (Clusters 2 and 10). In contrast, some miRNAs appeared to be down-regulated only in FP3 of WXS (F) (Cluster 4). The differential expression of these miRNAs (Cluster 4) suggests that they may be related to male sterility during fertility transition. Their high expression levels during the meiosis stage (SP3 and FP3) might inhibit some genes with essential roles in anther development. Eight known miRNAs (miRNA156a-j, miRNA3979, miRNA159c/d/e, miRNA171a/c/e/i, miRNA398b, miRNA164d, miRNA528, and miRNA408) were selected to authenticate expression profiles. The results showed that in WXS (S), expression levels of miRNA156a-j and miRNA171a/c/e/i were down-regulated (Figure 4B), whereas the rest of these miRNAs were up-regulated. These results were consistent with the high-throughput sequencing data, indicating that the results of this study were reliable.

### Validation of the miRNA expression analysis and their targets by qPCR

**Figure 5:**
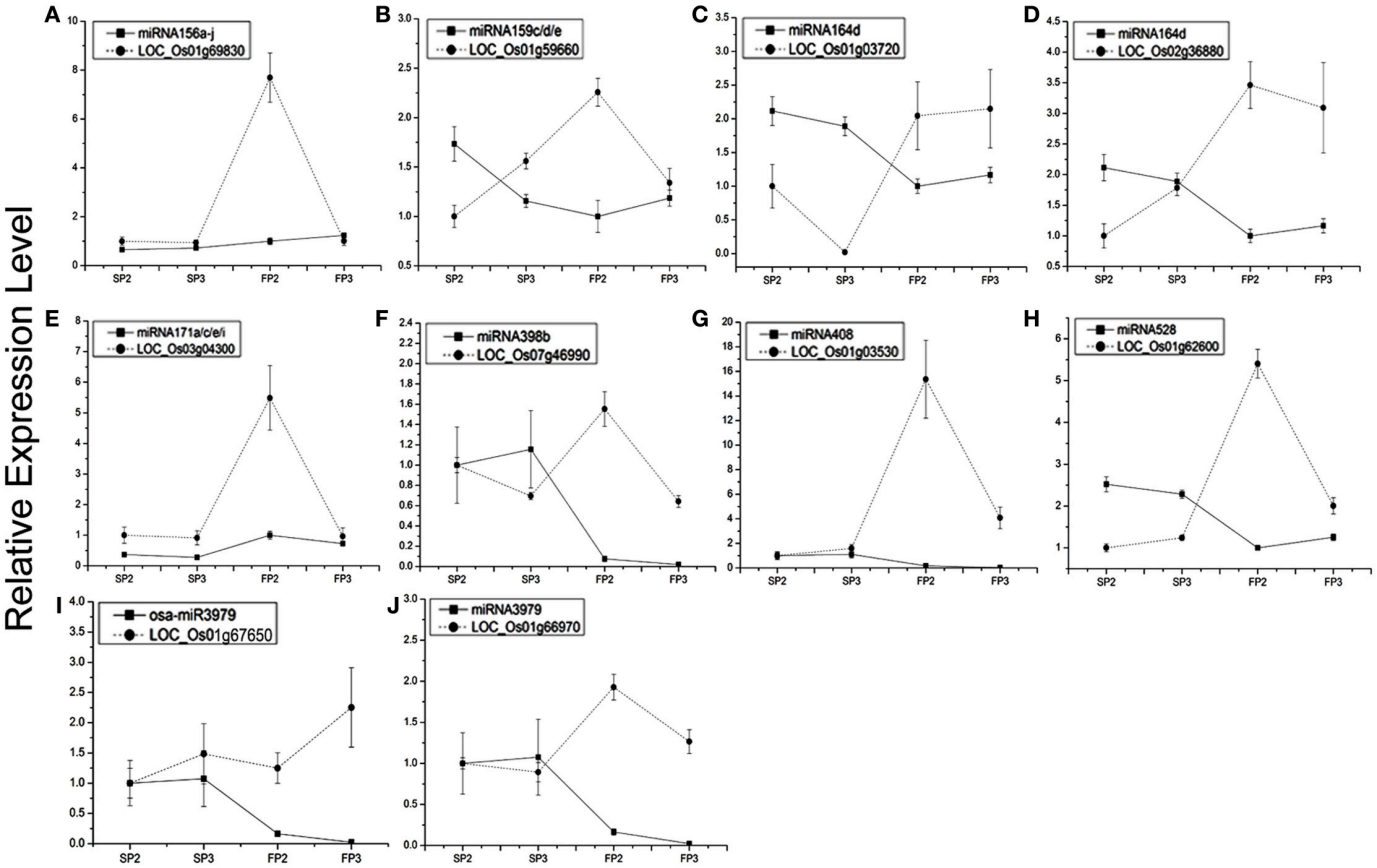
**Expression profiling analysis of several miRNAs and their corresponding target genes**. Actin was used as a reference for the target genes. The error bars indicate the standard deviation of three replicates. **(A)** miRNA156a-j and its target LOC_Os01g69830. **(B)** miR159c/d/e and its target LOC_Os01g59660. **(C)** miR164d and its target LOC_Os01g03720. **(D)** miR164d and its target LOC_Os02g36880. **(E)** miR171a/c/e/i and its target LOC_Os03g04300. **(F)** miR398b and its target LOC_Os07g46990. **(G)** miRNA408 and its target LOC_Os01g03530. **(H)** miR528 and its target LOC_Os01g62600. **(I)** miR3979 and its targets LOC_Os01g67650. **(J)** miR3979 and its target LOC_Os01g66970.

To study the correlation between the miRNAs and their targets, the 10 miRNA targets were examined by qPCR analysis (Figures 5A–J). We found that the expression of six miRNAs was negatively correlated with the expression of their targets. These miRNA targets were miRNA156a-j-OsSPL2 (LOC_Os01g69830, Figure 5A), miR159c/d/e-MYB (LOC_Os01g59660, Figure 5B), miR164d-No apical meristem protein (LOC_Os02g36880, Figure 5C), miR398b-copper/zinc superoxide dismutase (LOC_Os07g46990, Figure 5F), miR528-laccase (LOC_Os01g62600, Figure 5H), and miR3979- zinc finger protein (LOC_Os01g66970, Figure 5J).

Our results also showed that the MYB transcription factor (LOC_Os01g59660) and No apical meristem protein (LOC_Os02g36880) were both predicted to be targets of osa-miR164d. However, the expression patterns of the two targets were different during the process of fertility transition. Transcripts of LOC_Os01g03720 were positively correlated with the expression of miR164d (Figure 5C), whereas the expression level of LOC_Os02g36880 was negatively correlated (at near-significant levels) with miR164d (Figure 5D). The results suggest that the expression levels of different targets of the same miRNA might be regulated differently. Some negative correlations were also found between expression levels of target genes and their corresponding miRNAs in WXS (S) and WXS (F), indicating that miRNA-mediated mRNA silencing occurred during anther development in the fertility transition.

### miRNA target prediction and functional analysis

**Figure 6:**
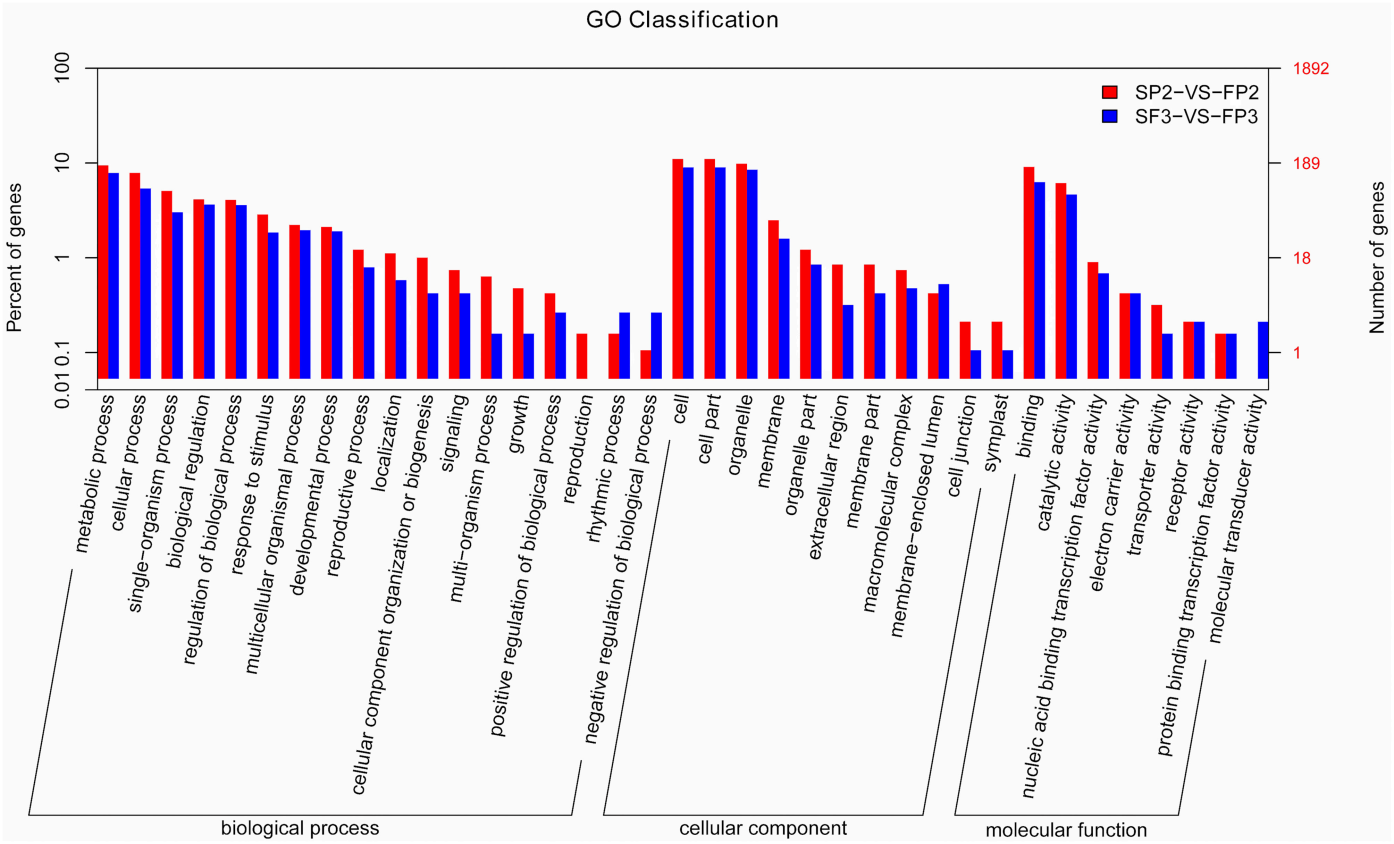
**Significant GO terms for the differentially expressed known miRNA targets**.

In plants, miRNAs regulate gene expression by interacting with their targets. Identifying the candidate genes targeted by the miRNAs would contribute to our understanding of the biological functions of miRNAs. Using psRNATarget, we identified 5192 targets for 435 known miRNAs, for an average of 11.9 targets per miRNA in this study (Table S6). We also identified multiple targets for the miRNAs at the 3′ terminal, such as Nramp6 for miR156c-3p and OsFBDUF58 for miR160a-3p, etc. As previously described, most of the predicted miRNA targets are particular transcription factors. We also identified some targets other than these conserved targets for conserved miRNAs. For example, besides NAC factors, osa-miR164 also targets the No apical meristem protein (Table S6). Predicted targets consist mainly of transposon proteins, retrotransposon proteins, growth-regulating factors, MYB family transcription factors, F-box domain-containing proteins, MADS-box family proteins, and SBP-box gene family members (Table S6). Targets of novel miRNAs were also predicted and show a much broader range of potential functions. In addition to transcription factors, various enzymes, transposon proteins, and unidentified expressed proteins were also targeted by novel miRNAs (Table S7). Based on the GO annotations (Figure 6) from this study, targets were enriched for the “metabolic process,” “cellular process,” and “single-organism process” terms of the “biological process” cluster. “Cell”, “cell part,” and “organelle” were the three most abundant “cellular component” terms. In the “molecular function” cluster, the top three terms were “binding,” “catalytic activity,” and “nucleic acid binding transcription factor activity” (Figure 6).

For the predicted targets of the 72 differentially expressed miRNAs (SP2 vs. FP2 or SP3 vs. FP3), an enrichment analysis was performed using AgriGO, and the 1892 predicted genes were categorized into 93 significant GO terms (*P* < 0.1) (Figure S3 and Table S8). Across these terms, “regulating” (GO: 0006355) was dominant within the main category of biological processes, and “regulation of RNA metabolic process” (GO: 0051252) was also found to be statistically significant in the same category (Table S8). Additionally, a high percentage of target genes was involved in “oxidoreductase activity” (GO:0052716, GO:0016682, and GO:0008447), “transferase activity” (GO:0046922, GO:0016433, and GO:0000179) and “binding” (GO:0005507, GO:0046914, and GO:0046872). GO terms related to various biological processes, including “regulation of gene expression” (GO: 0010468), “phenylpropanoid catabolic process” (GO: 0046271), “lignin metabolic process” (GO: 0009808), “rRNA modification” (GO: 0000154), “transcription” (GO: 0045449), “regulation of long-day flowering photoperiodism” (GO: 0048586), and “regulation of pollen tube growth” (GO: 0080092), were also found to be significantly enriched among the known miRNAs (Figure S3, and Table S8).

### Observation of the miRNA editing events

**Figure 7:**
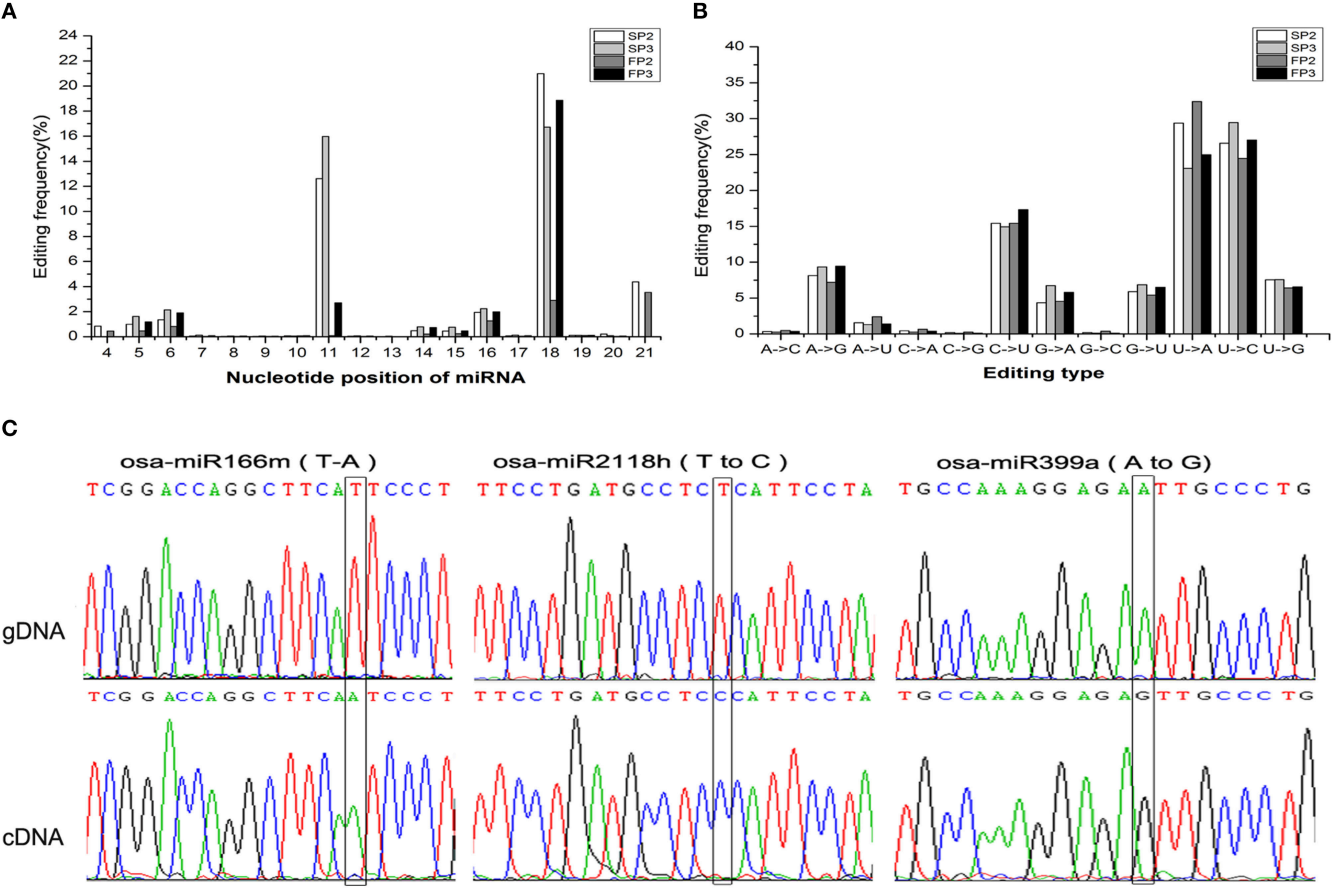
**Observation of the miRNA editing events. (A)** Summary of the nucleotide substitution types among the miRNAs observed in each library. **(B)** Summary of the nucleotide substitution positions among the miRNAs observed in each library. **(C)** Validation of the editing sites inferred from deep sequencing via Sanger sequencing. The edited positions are highlighted with black frames. The top trace is genomic DNA (gDNA), and the bottom trace is cDNA.

RNA editing is another post-transcriptional modification that generates divergence between RNAs and their genomic DNA sequences. Previous studies have documented that miRNA editing occurs in rice during the grain-filling stages (Yi et al., 2013). In this study, a large number of miRNA editing events were found during fertility transition (Table S9). These miRNA editing events mainly occurred at nucleotide positions 11 and 18 from the 5′ terminus (Figure 7A). The most abundant nucleotide substitution was U to A, which accounted for over 30% of substitutions in the SP2 and FP3 libraries; this was followed by the U to C type substitution, which comprised more than 25% (Figure 7B). These results were similar to those reported by Yan et al. (2015).

To validate the occurrence of miRNA editing, three editing types (U to A, U to C, and A to G) were examined in osa-miR166m, osa-miR2118h, and osa-miR399a, respectively. Using PCR amplification, precursor miRNA sequences from genomic DNA and mature miRNA sequences from cDNA were cloned to characterize nucleotide substitutions. This sequence comparison analysis further confirmed that miRNA editing events were reliably detectable during miRNA generation (Figure 7C).

## Discussion

Previous reports have studied the relationship between miRNAs and CMS in maize (Shen et al., 2011), cotton (Wei et al., 2013), *Brassica juncea* (Yang et al., 2013), and rice (Yan et al., 2015). However, few studies have investigated miRNAs from rice PTGMS lines. Therefore, characterizing the role of miRNAs in rice PTGMS lines would be extremely useful and could contribute to an improved understanding of the molecular functions of miRNAs during fertility transition in the male sterile rice lines used in two-line systems. In this study, we examined miRNA profiles determined by high-throughput sequencing and qPCR to investigate the expression of miRNAs in the rice PTGMS line WXS. These analyses revealed that miRNAs targeted many transcription factors associated with metabolism and signal transduction and play important roles in anther development. In this study, we found a total of 26 conserved miRNAs that were differentially expressed between WXS (S) and WXS (F) during fertility transition (Table 2). Of these, 11 miRNAs were down-regulated and 15 miRNAs were up-regulated. Of these, several members of the miR156 family showed significant differential expression levels in the four libraries (Table 2). It was apparent that expression levels of miRNAs were higher in WXS (F) than in WXS (S) during fertility transition.

In rice, it is well known that miR156 targets SBP-box gene family (SPL) proteins, which play important roles in the proper development of sporogenic tissues. Previous studies have reported that miR156 regulates the timing of flower formation through its target SPL3, which activates the expression of APETALA1 (Yamaguchi et al., 2009). In the present study, the low expression levels of miRNA156 found in WXS (S) might affect flower formation during pollen development. Many studies have reported that the formation of pollen exine is important for pollen development (Qin et al., 2013). In loblolly pine, laccase genes were reported to be expressed and involved in plant pollen development (Turlapati et al., 2011). The osa-miR3979 target laccase was found to participate in regulating the pollen abortion process in a maize CMS line (Shen et al., 2011). However, in the present study, osa-miR528 was predicted to target the laccase precursor protein (LOC_Os01g62600). Furthermore, our qPCR analysis confirmed that the expression of osa-miR528 is negatively correlated with that of its target laccase (LOC_Os01g62600) (Figure 5H). These results indicated that osa-miR528, which targets laccase, might be involved in the fertility transition of WXS. In all eukaryotes, the ubiquitous, multifunctional calcium sensor calmodulin (CaM) mediates calcium action by regulating the activity and function of many proteins. Calcium and CaM play crucial roles in pollen germination and pollen tube growth. In Arabidopsis, a calmodulin-binding protein was reported to be essential for pollen germination (Golovkin and Reddy, 2003). Previous studies have also documented that osa-miR1432 and osa-miR812d may be involved in Ca^2+^-mediated signaling pathways by targeting genes that encode EF-hand family proteins and CaM/Ca-dependent protein kinase, respectively (Yan et al., 2015). In our study, osa-miR5976 was predicted to target a gene coding for a calmodulin binding protein (LOC_Os12g36940), and expression of osa-miR5976 was up-regulated in WXS (S) (Table 2).

The conserved targets of miR159 are MYB transcription factors, which have been reported to be involved in flower development and are essential for fertility (Jones et al., 2006; Tsuji et al., 2006). In rice, mutations in OsGAMYB have resulted in defects in anther and pollen development, and overexpression of osa-miR159 leads to male sterility (Kaneko et al., 2004; Tsuji et al., 2006). In this study, three members of osa-miR159 (osa-miR159c/d/e) were found to be up-regulated in WXS (S) (SP2 and SP3). The expression levels of osa-miR159 and its target, GAMYB transcription factor (LOC_Os01g59660), were confirmed by qPCR, which showed that these miRNAs and their targets were negatively correlated (Figure 5B). Therefore, it is highly likely that osa-miR159 silenced the expression of MYB proteins, which affect anther development in WXS (S). Moreover, relatively high expression levels of osa-miR164d were found in the meiosis stage of WXS (S) (SP3). The gene coding for the transcription factor NAC is considered to be the target of osa-miR164. In many plant species, NAC transcription factors are involved in plant tolerance to biotic or abiotic stresses. The SP2 to SP3 stages in WXS (S) are important for fertility transition during anther development. Up-regulated expression of osa-miR164d in WXS (S) (SP3) indicated that it may be involved in fertility transition. Moreover, osa-miR444f was predicted to target the gene encoding a pentatricopeptide repeat (PPR) protein (LOC_Os04g14450). PPR proteins are a large family with tandem repeats of a degenerate 35 amino acid motif. In higher plants, PPR proteins can suppress the expression of mitochondrial genes associated with cytoplasmic male sterility to restore fertility (Desloire et al., 2003; Wang et al., 2004). In our study, the higher expression of osa-miR444f in WXS (S) may silence the expression of PPR proteins, ultimately affecting their functions in anther development. Furthermore, previous reports have also shown that accumulation of two Arabidopsis miRNAs (miR171, miR398) oscillates during the diurnal cycle: increasing during the light period and decreasing in darkness (Christelle et al., 2009). The miRNA171c targets SCL6-II, SCL6-III, and SCL6-IV regulate shoot branching in Arabidopsis (Wang et al., 2010; Manavella et al., 2013). In this study, miRNA398b was down-regulated in WXS (S) (Table 2). However, miRNA171, which showed higher expression in WXS (F), may target an ankyrin repeat domain-containing protein (ARDCP, LOC_Os03g04300).

**Figure 8:**
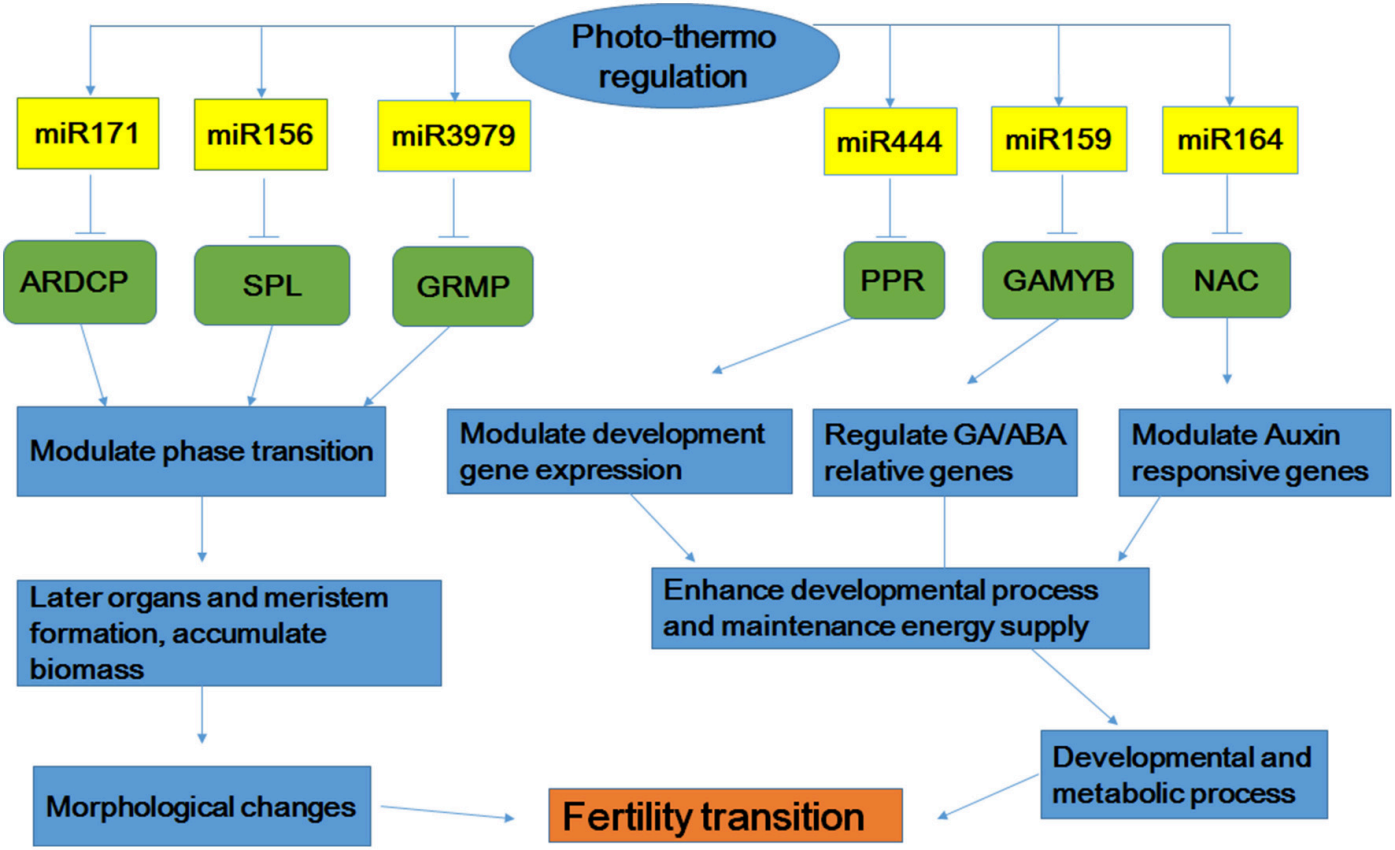
**Possible microRNAs-dependent regulatory pathways that participate in fertility transition**. ARDCP, Ankyrin repeat domain containing protein; SPL, OsSPL-SBP-box gene family member; GRMP, gibberellin response modulator protein; PPR, PPR protein; GAMYB, MYB family transcription factor.

Based on the comprehensive analysis described above, we proposed a possible model by which miRNAs control the signaling pathways involved in fertility transition of the rice PTGMS line WXS (Figure 8). MiR171, miRNA156, and miRNA3979 interact with their respective target genes (ARDCP, SPL, and GRMP) to modulate phase transition. These interactions then result in the subsequent formation of organs and the meristem and the accumulation of biomass, and finally result in morphological changes to the rice anther. Moreover, the interactions of miRNA444, miRNA159 and miRNA164 with their corresponding target genes (PPR, GAMYB, and NAC) modulate the expression of developmental genes, GA/ABA-related genes, and auxin-responsive genes to promote the transmission of signals that enhance developmental processes and maintain energy supply. These properties affect development and metabolic processes. Fertility transition in the rice PTGMS line WXS may occur due to combined action of the regulatory interactions described above. Although fertility transition in rice PTGMS lines is a highly complex process, it is certain that miRNAs must play very important roles in the regulation of rice anther development. This study also showed that miRNAs may play a critical role in the fertility transition stage, which adapts to variations in temperature and photoperiod. Our functional description and target analysis of miRNAs provide more clues for understanding the different ways gene expression is regulated during fertility transitions. These miRNA profiles also revealed an important component of the gene regulatory circuit and may provide insights for further investigations of PTGMS rice lines.

## Conclusion

In this study, we performed high-throughput sequencing to identify the miRNA expression profiles of the rice PTGMS line WXS during fertility transition. A total of 497 known and 273 novel miRNAs were identified, and some of these miRNAs were validated by stem-loop RT-PCR analysis. MiRNA editing events were also observed and validated during anther development. Target prediction analysis indicated that some miRNAs are involved in anther development and male sterility in the WXS line. The characterization and comparative expression profiling of the miRNA transcriptome in this study lays the foundation for understanding the complex miRNA-mediated regulatory networks in rice anther development. A possible model for the control that miRNAs exert on signaling pathways during fertility transition in the rice PTGMS line WXS was proposed. Further functional studies on differentially expressed miRNAs will provide a better understanding of miRNA-mediated regulation mechanisms during fertility transitions in rice PTMGS lines.

## Author contributions

YD and HZ conceived and designed the experiments. HZ, QQ, HC, JJ performed the experiments. HZ and JH analyzed the data and drafted the manuscript. YD contributed reagents/materials/analysis tools and modified the manuscript. All of the authors carefully checked and approved this version of the manuscript.

## Data access

The miRNA raw data released have been deposited at NCBI in the Gene Expression Omnibus (GEO) database under the accession number: GSE74003 (http://www.ncbi.nlm.nih.gov/geo/query/acc.cgi?acc=GSE74003).

## Funding

This study is supported by National Natural Science Foundation of China (31471464), and “973” Program of China (2013CB126900).

### Conflict of interest statement

The authors declare that the research was conducted in the absence of any commercial or financial relationships that could be construed as a potential conflict of interest.

## Supplementary material

Table S1 **Primers used in this study**.

Table S2 **The filtering for small RNA clean reads in the four libraries of WXS**.

Table S3 **Summary of the known miRNAs identified in the four libraries of WXS**.

Table S4 **Summary of the novel miRNAs predicted in the four libraries of WXS**.

Table S5 **Comparison of the expression levels for the known miRNAs between WXS (S) and WXS (F) (SP2—FP2 and SP3—**FP3**)**.

Table S6 **Targets predicted for the known miRNAs**.

Table S7 **Targets predicted for the novel miRNAs**.

Table S8 **Significant GO terms for the differentially expressed known miRNA targets**.

Table S9 **Summary of the miRNA editing types, positions, SNP counts and editing rates in the four libraries (SP2, SP3, FP2, and FP3)**.

Figure S1 **Fragment size distributions and first nucleotides of the novel miRNAs**. The fragment size distribution analysis indicated that most of the miRNAs have nucleotide lengths of 21 nt **(A)**. The first nucleotide analysis of these novel miRNAs showed that the bases rates of U, C, A, and G appeared at frequencies of 49, 26, 17, and 9%, respectively **(B)**.

Figure S2 **Scatter plots obtained by comparison of the expression patterns of miRNAs identified between SP2 and FP2 (A) and between SP3 and FP3 (B)**.

Figure S3 **Gene Ontology (GO) analysis of the differentially expressed miRNA target genes of WXS via AgriGO**. Target transcripts of differentially expressed miRNAs under low temperatures and shorter light periods compared with the control (natural conditions) were designated as photo-thermo responsive genes. The box colors indicates levels of statistical significance: blue = 0.05; green = e-05; yellow = e-10; and orange = e-15. Brown indicates genes associated with the particular GO term in the rice database.
