# Supplementary figures and images for: Small RNA Profiles of the Rice PTGMS Line Wuxiang S Reveal miRNAs Involved in Fertility Transition
Source: Front Plant Sci. 2016 Apr 20;7:514. doi: 10.3389/fpls.2016.00514 (PMC4837141; doi:10.3389/fpls.2016.00514)

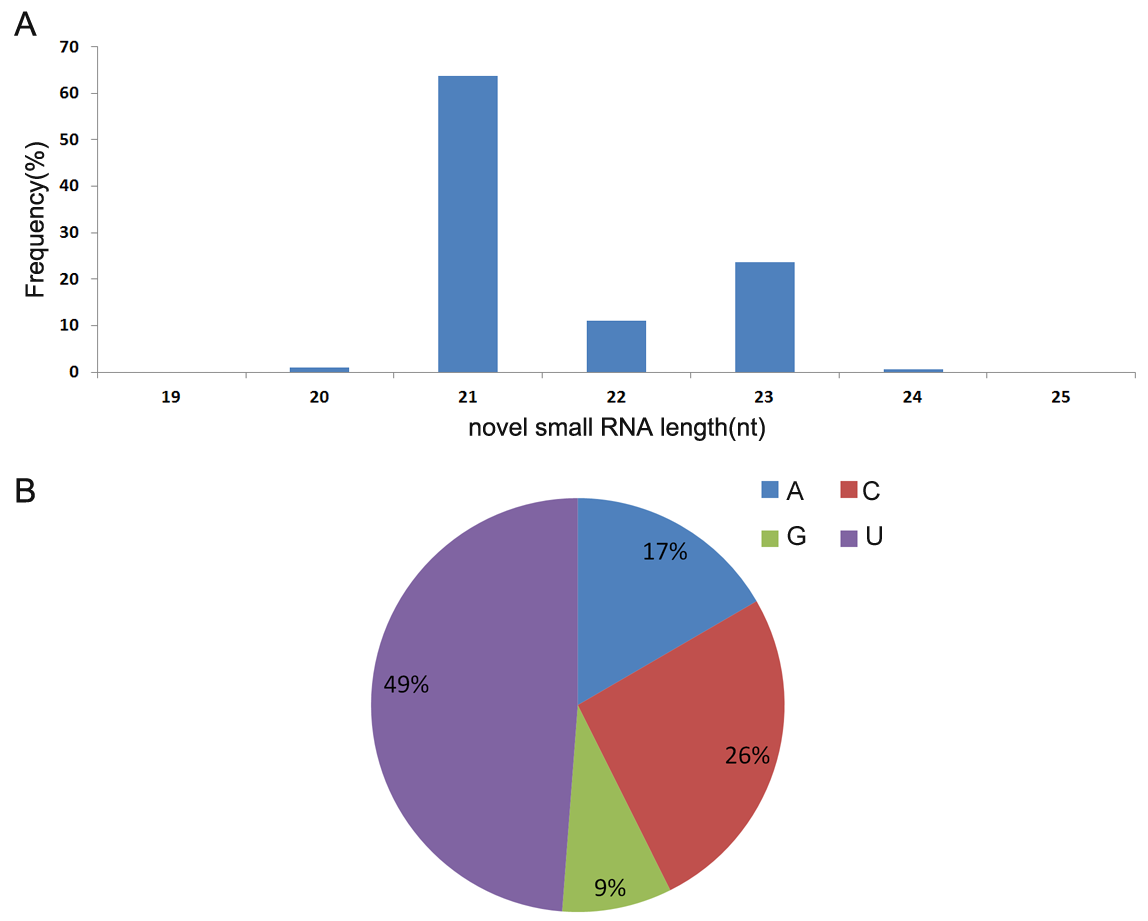

Supplement: Figure S1 — Fragment size distributions and first nucleotides of the novel miRNAs. The fragment size distribution analysis indicated that most of the miRNAs have nucleotide lengths of 21 nt (A). The first nucleotide analysis of these novel miRNAs showed that the bases rates of U, C, A, and G appeared at frequencies of 49, 26, 17, and 9%, respectively (B). [file Image1.TIF]

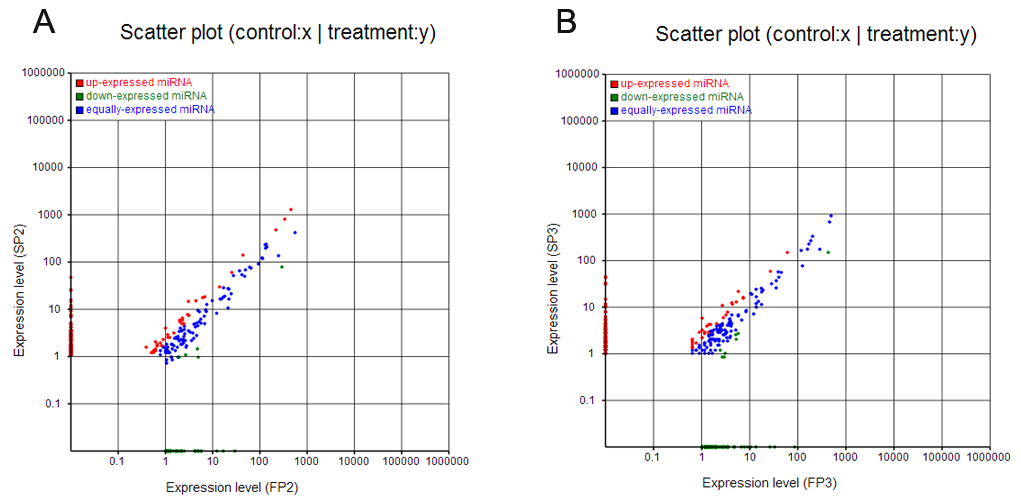

Supplement: Figure S2 — Scatter plots obtained by comparison of the expression patterns of miRNAs identified between SP2 and FP2 (A) and between SP3 and FP3 (B). [file Image2.TIF]

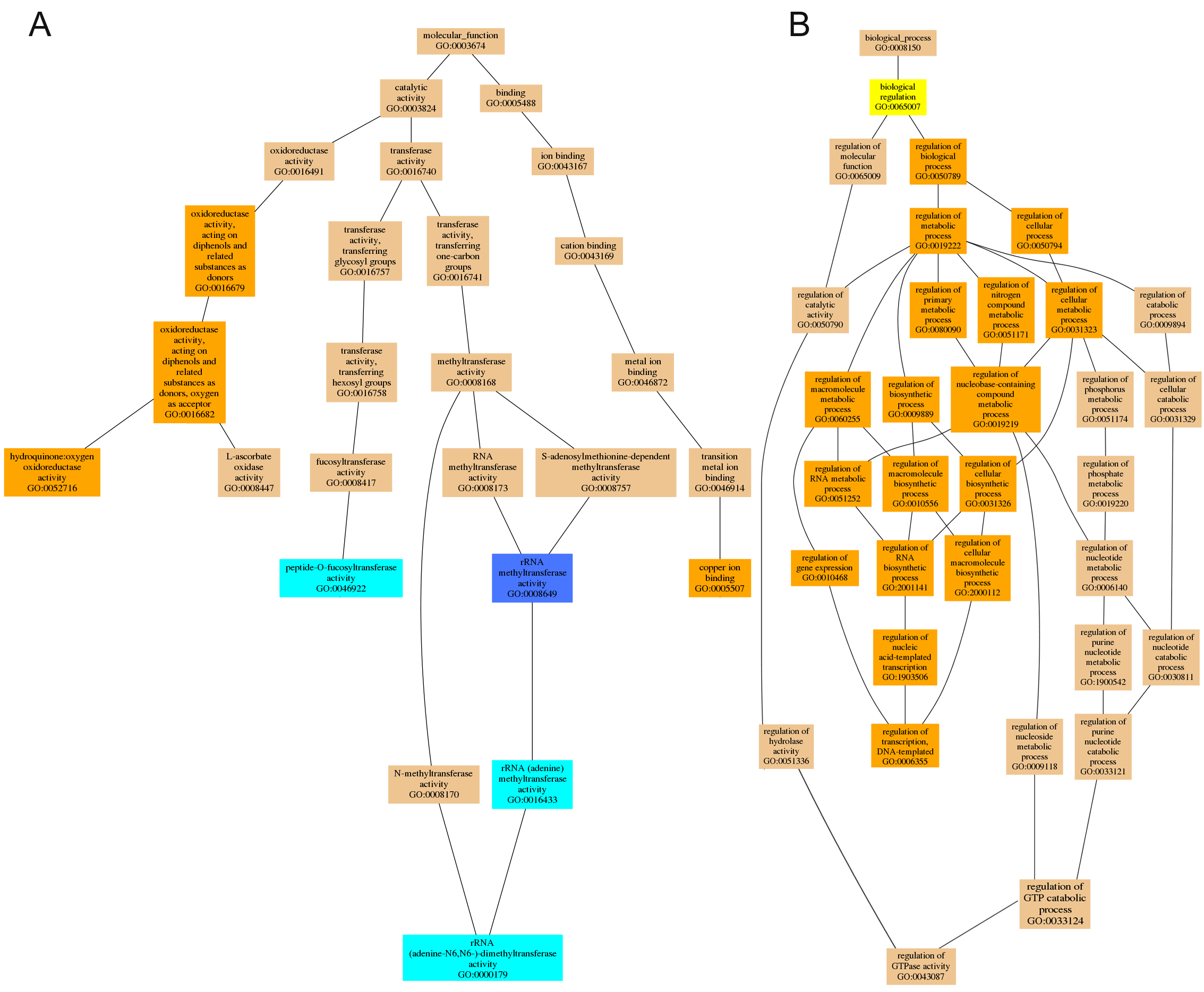

Supplement: Figure S3 — Gene Ontology (GO) analysis of the differentially expressed miRNA target genes of WXS via AgriGO. Target transcripts of differentially expressed miRNAs under low temperatures and shorter light periods compared with the control (natural conditions) were designated as photo-thermo responsive genes. The box colors indicates levels of statistical significance: blue = 0.05; green = e-05; yellow = e-10; and orange = e-15. Brown indicates genes associated with the particular GO term in the rice database. [file Image3.TIF]
